# Design of the Growth hormone deficiency and Efficacy of Treatment (GET) score and non-interventional proof of concept study

**DOI:** 10.1186/s12902-018-0237-3

**Published:** 2018-02-13

**Authors:** Peter H. Kann, Simona Bergmann, Martin Bidlingmaier, Christina Dimopoulou, Birgitte T. Pedersen, Günter K. Stalla, Matthias M. Weber, Stefanie Meckes-Ferber

**Affiliations:** 1 Division of Endocrinology & Diabetology, Philipp’s University Marburg, D-35033 Marburg, Germany; 2 Endocrine Laboratory, Medizinische Klinik und Poliklinik IV, Ludwig-Maximilians University, 80336 Munich, Germany; 3 Neuroendocrinology, Max-Planck-Institute for Psychiatry, 80804 Munich, Germany; 4 Epidemiology, Novo Nordisk A/S, 2860 Søborg, Denmark; 5 Endocrinology & Metabolism, Johannes Gutenberg University Hospital, 55131 Mainz, Germany; 6 Clinical, Medical & Regulatory Department, Novo Nordisk Pharma GmbH, 55127 Mainz, Germany

**Keywords:** Clinical study, Growth hormone, Growth hormone deficiency, Quality of life

## Abstract

**Background:**

The adverse effects of growth hormone (GH) deficiency (GHD) in adults (AGHD) on metabolism and health-related quality of life (HRQoL) can be improved with GH substitution. This investigation aimed to design a score summarising the features of GHD and evaluate its ability to measure the effect of GH substitution in AGHD.

**Methods:**

The Growth hormone deficiency and Efficacy of Treatment (GET) score (0–100 points) assessed (weighting): HRQoL (40%), disease-related days off work (10%), bone mineral density (20%), waist circumference (10%), low-density lipoprotein cholesterol (10%) and body fat mass (10%). A prospective, non-interventional, multicentre proof-of-concept study investigated whether the score could distinguish between untreated and GH-treated patients with AGHD. A 10-point difference in GET score during a 2-year study period was expected based on pre-existing knowledge of the effect of GH substitution in AGHD.

**Results:**

Of 106 patients eligible for analysis, 22 were untreated GHD controls (9 females, mean ± SD age 52 ± 17 years; 13 males, 57 ± 13 years) and 84 were GH-treated (31 females, age 45 ± 13 years, GH dose 0.30 ± 0.16 mg/day; 53 males, age 49 ± 15 years, GH dose 0.25 ± 0.10 mg/day). Follow-up was 706 ± 258 days in females and 653 ± 242 days in males. The GET score differed between the untreated control and treated groups with a least squares mean difference of + 10.01 ± 4.01 (*p* = 0.0145).

**Conclusions:**

The GET score appeared to be a suitable integrative instrument to summarise the clinical features of GHD and measure the effects of GH substitution in adults. Exercise capacity and muscle strength/body muscle mass could be included in the GET score.

**Trial registration:**

NCT number: NCT00934063. Date of registration: 02 July 2009.

**Electronic supplementary material:**

The online version of this article (10.1186/s12902-018-0237-3) contains supplementary material, which is available to authorized users.

## Background

Growth hormone (GH) is a pleiotropic hormone. Whereas growth failure is the relevant symptom of childhood GH deficiency (GHD), adult GHD (AGHD) is a recognised syndrome with adverse phenotypic, metabolic and health-related quality of life (HRQoL) features [1], which improve in many patients when GH is substituted [2, 3]. For some chronic diseases with multiple clinical facets and complications (e.g., diabetes), a composite of clinical endpoints has been defined as a primary outcome measure for study purposes to evaluate the effect of therapeutic interventions [4, 5].

The objective of this project was to design and, in a second step, conduct a non-interventional proof of concept study to evaluate an instrument that allows quantification and summarising of the various facets of AGHD and the therapeutic response to GH replacement. The composite score was given the acronym “GET – Growth hormone deficiency and Efficacy of Treatment” and aimed to provide a quantitative integrative picture of parameters that, based on evidence in the literature [2, 3], are considered clinically, economically and socially relevant in a large population.

All the parameters chosen to be integrated into the GET score had previously been shown, according to the criteria of evidence-based medicine (EBM), to be affected by GHD and to be improved following GH substitution in AGHD. The weighting of parameters in the GET score was arbitrarily defined by the study group according to their estimated clinical relevance (experts’ opinion). The parameters are all assessed in routine clinical practice and the composite score was intended to have the potential to be used for scientific purposes as well as in clinical practice. In the second step of this project, a prospective, non-interventional, multicentre proof of concept study was performed to investigate whether the GET score was able to distinguish between untreated and GH-treated patients with AGHD. Based on existing knowledge regarding the effect of GH replacement in patients with AGHD on the parameters included in the score, the difference between control and treated patients over 2 years was expected to be 10 points, and this difference was assumed to be clinically relevant (see details in Methods). If this was shown, the GET score would be considered a scientifically useful and clinically relevant instrument. In addition, the effect of GH therapy on insulin-like growth factor I (IGF-I) standard deviation score (SDS) and on the individual clinical parameters comprising the GET score was evaluated.

## Methods

### GET score assessment and definition

GET score items were selected according to evidence available in the literature [2, 3], and their weighting was defined arbitrarily following extensive discussion in the study group. The GET score was designed to cover a range between 0 and 100 points, composed from clinically measurable parameters. It was intended that GH-untreated patients with AGHD should be positioned approximately in the middle of the range (with a mean of ~ 50 points and a standard deviation [SD] of ~ 20 points) and that the range should allow the measurement of treatment effects. Fifty percent of the GET score points were generated from HRQoL parameters and 50% from physical measurements of somatic parameters.

The Short-Form Health Survey 36 (SF-36), one of the most commonly used generic instruments for measuring HRQoL, covers eight HRQoL elements assessing physical and psychological health [6]. Previous research has established the relationship between the EuroQol five dimensions questionnaire (EQ-5D), a generic five-item instrument providing a simple descriptive profile and a single index value for health status, and a tool used to measure HRQoL in patients with AGHD [7, 8]. An increase in the SF-36 and EQ-5D visual analogue scale (VAS) score reflects an improvement in self-perceived health. The SF-36 and the VAS component of EQ-5D (EQ-5D-VAS) have both been used previously in patients with AGHD [7, 9]. The QoL-Assessment of GHD in Adults (QoL-AGHDA), a disease-specific, need-based measure [10], developed based on in-depth interviews with adult patients with GHD is also a recognised measure for the assessment of QoL. However, restricted licence use did not permit use of this tool in our study.

The HRQoL parameters of the GET score comprised the SF-36 score [7] (20 points) and the EQ-5D-VAS (20 points), together with the disease-related days off work (10 points).

Details on the allocation of the GET score points from SF-36 and EQ-5D-VAS are given in Additional file 1: Table S1. As the SF-36 covers eight dimensions, the arithmetic mean of the score points from each dimension was taken and included into the GET score (an example is shown in Additional file 1: Table S2). Based on data from Saller et al., [11] > 30 disease-related days off work during the previous 6 months generated a score of 0 points, and < 4 disease-related days off work generated a score of 10 points (Additional file 1: Table S1).

The somatic parameters comprised bone mineral density (BMD) (20 points), waist circumference (10 points), low-density lipoprotein cholesterol (LDL-C) (10 points), and body fat mass (10 points). Details on the allocation of the GET score points for the somatic parameters are provided in Additional file 1: Table S3.

Dual-energy X-ray absorptiometry (DXA) is the gold standard for BMD measurement [3, 12]. As patients’ ages spanned more than five decades, the z-score was selected as the most suitable parameter for measuring BMD. The most pronounced effect of GH substitution on BMD is detectable at the lumbar spine [13], hence this was the measuring site for the GET score. Based on published data [14], DXA BMD lumbar spine z-score ≤ −2 was assigned a score of 0 points, and a z-score ≥ 0 was assigned a score of 20 points.

Waist circumference reflects visceral fat accumulation and is established as a key criterion for the diagnosis of metabolic syndrome and as an independent cardiovascular risk factor [15]. When including this parameter in the GET score, individual variance, risk threshold, and published data from patients with AGHD with rather small therapeutic effects had to be considered [16, 17]. Therefore, waist circumference ≥99 cm in females / ≥113 cm in males scored 0 points, and waist circumference ≤ 80 cm in females / ≤94 cm in males scored 10 points.

Based on the baseline values and the therapeutic effects of GH substitution on LDL-C in patients with AGHD [18, 19], LDL-C ≥ 3.98 mmol/L (154 mg/dL) scored 0 points, and ≤2.59 mmol/L (100 mg/dL) scored 10 points.

Using a Tanita scale, body fat mass can be assessed with body impedance analysis. Based on data from Rosenfalck et al. [20], body fat mass percentage ≥ 44.1% scored 0 points, and ≤21.5% scored 10 points.

To calculate a GET score, the first step is to calculate the overall SF-36 GET score points by taking the average of all eight SF-36 GET score points based on the transformed SF-36 domain scores (Additional file 1: Table S1). The second step is to add the GET score points for the remaining HRQoL parameters – EQ-5D-VAS and disease-related days off work (Additional file 1: Table S1). The third step is to look up the GET score points for the somatic parameters using the GET score points as shown in Additional file 1: Table S3. The addition of all components sums up to the final GET score. An example of a calculation of GET score is provided in Additional file 1: Table S4. If individual parameters are missing, the score is calculated without these parameters, but adjusted accordingly (Additional file 1: Table S5). For example, BMD has a weighting of 20%; the maximum score achievable without BMD would be 80. If a patient achieved a determined score of 67 without BMD, adjustment of the determined score would be 67/80*100, resulting in a final GET score of 83.75 (Additional file 1: Table S5). A minimum number of parameters giving a total weighting of ≥70% is required to determine the adjusted GET score, otherwise the GET score is set to missing.

### Proof of concept study

#### Study design

GH-treatment-naïve patients with AGHD, defined according to GH Research Society criteria [3], under the care of endocrinologists, were enrolled into a prospective, observational, non-interventional, multicentre proof of concept study.

The indication and clinical decisions regarding GH replacement (Norditropin® [somatropin, recombinant human GH], Novo Nordisk A/S, Denmark) were made by the treating physician according to usual clinical practice. GH-treated patients were compared with patients in whom no treatment was initiated; the decision not to initiate GH replacement was taken jointly by the patient and the physician. The study was performed in accordance with the Declaration of Helsinki [21]. Ethical permissions were obtained from the Ethical Commission of the Chamber of Physicians of the German Federal State of Hessia. Informed consent was obtained from all study participants.

The study recruitment period was originally planned for 24 months, but extended to 36 months due to limited recruitment. Participation commenced at visit 1, when baseline data were collected and GH treatment was initiated in the treatment group. Interim follow-up visits (visits 2–4) were planned for approximately every 6 months, but occurred at varying intervals, and the participants’ involvement concluded at visit 5. If the patient prematurely discontinued participation, the last interim visit became the final visit. Duration of follow-up was calculated as days between first and last visit.

The inclusion criteria for data analysis were availability of baseline demographic data (gender, date of birth), information about GH therapy for treated patients and at least one of four follow-up visits.

The GET score was calculated, and if there were too few parameters to provide a total weighting of ≥70%, the GET score was set to missing. In the proof of concept study, IGF-I concentrations were measured mainly as a parameter for plausibility, verifying whether GH had or had not been administered. IGF-I was assessed centrally using the iSYS automated chemiluminescent IGF-I assay (Immunodiagnostic Systems Ltd., Boldon, UK). The assay employs two monoclonal antibodies and is calibrated against WHO International Standard 02/254 (National Institute for Biological Standards and Control, Hertfordshire, UK) [22].

#### Statistical analysis

In observational studies, clinical practice is reflected in missing values, missing visits and fewer untreated controls than treated patients, thereby providing unbalanced data; therefore, a repeated measures model was found to be the most appropriate method to analyse the available data. The study sample size was determined by the ability to recruit patients within the study period. By using the repeated measures multiple regression model for the GET score analysis, correlation of data within the individual patient were taken into account when patients were observed at several visits over time within the study period. Any overall differences between the mean GET score of the two groups in the full study period could be detected. Due to the ageing of the patients over the study period, deterioration over time could potentially occur in the parameters included in the GET score, therefore untreated controls versus treated patients were evaluated.

The model included treatment group (control and treated), visit, and the interaction term between visit and treatment as explanatory variables. Gender, age and treatment duration were also included in the model to adjust for potential differences in patient characteristics in the two groups. The overall difference in GET score between control and treated groups in the full study period was estimated by least squares means (LSM). Missing data were handled by the repeated measures model when evaluating the GET score and were considered missing completely at random. Descriptive statistics were applied for all parameters and data are presented as mean ± SD, unless otherwise stated. Statistical analysis was performed using SAS v9.4 (SAS Institute Inc., Cary, North Carolina, USA).

## Results

A total of 106 patients were eligible for analysis (controls: 9 females, 13 males; GH-treated: 31 females, 53 males). Baseline characteristics for all 106 patients and mean GH starting dose for treated patients are shown in Table 1. A baseline GET score could only be calculated for 75 patients due to missing data. In the follow-up evaluation of the GET score the 75 patients were distributed as 15 control (5 females, 10 males) and 60 GH-treated (22 females, 38 males) patients.

**Table 1:** Baseline demographics and characteristics of the included AGHD patients

Measurement	Female control group		Female treated group		Male control group		Male treated group	
	N	Mean ± SD	N	Mean ± SD	N	Mean ± SD	N	Mean ± SD
Age (years)	9	51.60 ± 16.76	31	44.86 ± 13.05	13	57.16 ± 12.88	53	48.73 ± 14.69
GH starting dose (mg/day)	9	0.00 ± 0.00	31	0.23 ± 0.13	13	0.00 ± 0.00	53	0.20 ± 0.09
IGF-I SDS	9	−1.13 ± 2.09	26	−1.40 ± 1.44	12	−1.58 ± 1.17	44	−1.28 ± 1.72
Diagnosis at baseline	9		31		13		53	
Acquired GHD (trauma)	1		1		0		1	
Acquired GHD (pituitary tumour)	4		9		7		25	
Acquired GHD (surgery/irradiation)	1		14		5		10	
Acquired GHD (other)	2		4		0		6	
Idiopathic GHD	1		0		0		5	
Hypopituitarism/pituitary abnormality	0		3		1		5	
Craniopharyngioma	0		0		0		1	

*AGHD* adults with growth hormone deficiency, *GET* Growth hormone deficiency and Efficacy of Treatment, *GH* growth hormone, *GHD* GH deficiency, *IGF-I* insulin-like growth factor 1, *N* number of participants eligible for analysis,*SD* standard deviation, *SDS* standard deviation score

At baseline, where all patients were in a GH-naïve stage, the overall mean ± SD GET score was estimated as 51.66 ± 20.48 score points, which was close to the intended mean baseline score of around 50 and intended SD of 20.

Baseline mean age was higher in the control versus the treated group and higher in males than females; however, the statistical model adjusted for this. Differences in age and gender did not reach a statistically significant level when included in the full repeated measures model evaluating the GET score.

Treatment (study) duration was longer for treated females (706.5 ± 258 days) versus treated males (653.6 ± 242 days). However, based on the results from the model, duration did not have a statistically significant effect on the GET score within the given study period.

### GET score

Mean unadjusted GET scores by gender at baseline and follow-up visits are shown in Table 2. Mean baseline GET scores were close to 50 in all groups (female controls: 51.14 ± 21.62; treated females: 47.02 ± 22.29; male controls: 49.78 ± 19.01; treated males: 54.92 ± 19.84).

**Table 2:** Mean^a^ GET score at baseline and follow-up visits by gender for GH-treated patients and controls

	GET score							
Visit	Female control group		Female treated group		Male control group		Male treated group	
	N	Mean ± SD	N	Mean ± SD	N	Mean ± SD	N	Mean ± SD
1 (baseline)	5	51.14 ± 21.62	22	47.02 ± 22.29	10	49.78 ± 19.01	38	54.92 ± 19.84
2	5	48.84 ± 13.26	19	51.45 ± 13.07	10	47.02 ± 13.76	33	57.68 ± 16.47
3	4	53.49 ± 10.96	19	52.48 ± 16.09	9	43.22 ± 18.31	32	60.97 ± 14.92
4	3	43.65 ± 24.66	12	47.60 ± 14.70	9	40.58 ± 11.79	24	58.57 ± 13.91
5	3	49.84 ± 23.29	11	47.59 ± 14.11	6	49.19 ± 17.32	29	56.03 ± 14.67

^a^Note the data presented are crude mean values and based on a variable number of patients

*GH* growth hormone,*GET* Growth hormone deficiency and Efficacy of Treatment, *N* number of participants in whom GET score was calculated, *SD* standard deviation

**Fig. 1:**
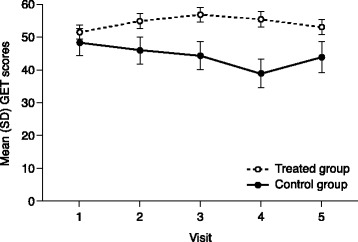
Estimated difference in the GET score between control and GH-treated groups during follow-up visits (EAS) EAS, effectiveness analysis set; GET, Growth hormone deficiency and Efficacy of Treatment; GH, growth hormone.

Fig. 1 shows the estimated GET scores for each group at every visit based on the repeated measures model. The analysis showed that GH treatment had an overall clinically relevant and statistically significant effect on the GET score of the expected magnitude, with a LSM difference of + 10.01 ± 4.01 (*p* = 0.0145) between the control and treated groups based on the full follow-up period in the study.

#### Changes in individual items contributing to the GET score

##### HRQoL

Improvements in HRQoL, as assessed with the SF-36, were observed in the GH-treated group for physical functioning (female + 4.64 ± 24.14 [*n* = 14]; male + 0.96 ± 14.42 [*n* = 26]), emotional role functioning (female + 5.36 ± 25.66 [*n* = 14]; male + 1.92 ± 30.49 [*n* = 26]) and physical role functioning (female + 12.05 ± 26.00 [*n* = 14]; male + 1.39 ± 20.17 [*n* = 27]); and in the female treated group only there were improvements in their general health perception (+ 10.93 ± 23.43 [*n* = 14]) and vitality (+ 9.82 ± 19.41 [*n* = 14]).

Using the EQ-5D-VAS, HRQoL was numerically higher at baseline in female controls (*n* = 6) (77.67 ± 25.32) versus treated females (*n* = 31) (58.71 ± 21.08), but numerically lower in male controls (*n* = 11) (56.36 ± 19.38) than treated males (*n* = 47) (65.15 ± 19.29). During the study period, mean EQ-5D-VAS score increased in GH-treated patients (mean change + 10.00 ± 11.73 females [*n* = 13]; + 6.38 ± 17.20 males [n = 26]). However, the score decreased substantially in female controls (*n* = 2) (mean change − 27.50 ± 3.54) and increased slightly in male controls (*n* = 6) (+ 4.33 ± 14.32). HRQoL assessed by the SF-36 was more variable than when assessed by the EQ-5D-VAS.

##### Disease-related days off work

The number of disease-related days off work during the previous 6 months varied throughout the study. There was a decrease at each visit for GH-treated female patients, and the change from baseline was − 30.00 ± 63.44 days by visit 5 (*n* = 5). There was no discernible pattern in the number of disease-related days off in the male treated group; number of days off was 8.21 ± 44.04 days below baseline at visit 5 (*n* = 19).

##### Bone mineral density

At baseline BMD assessed by DXA z-score was − 0.54 ± 1.42 in female controls (*n* = 5), − 0.20 ± 1.30 in male controls (*n* = 9), − 0.23 ± 1.06 in treated females (*n* = 9) and − 0.68 ± 1.86 in treated males (*n* = 20). There were small fluctuations throughout the study, with minimal change from baseline by visit 5: female controls (*n* = 2): + 0.05 ± 0.21; male controls (*n* = 3): + 0.27 ± 0.45; treated females (*n* = 4): + 0.18 ± 0.59; treated males (*n* = 9): + 0.49 ± 0.45. However, the small number of patients who underwent DXA analysis made these data difficult to interpret.

##### Waist circumference

During the study, waist circumference (cm) increased in controls (females [*n* = 6] + 2.67 ± 5.28; males [*n* = 5] + 3.86 ± 4.84) and decreased in GH-treated patients (change from baseline [cm]: female [*n* = 7] –2.86 ± 5.15; males [*n* = 20] –1.23 ± 7.55).

##### LDL-C

During the study, LDL-C increased slightly from baseline for female controls (*n* = 9) and all male patients (*n* = 12) (female controls + 0.40 ± 0.82 mmol/L [*n* = 5]; male controls + 0.04 ± 0.72 mmol/L [*n* = 7]; treated males + 0.01 ± 0.63 mmol/L [*n* = 23]) and decreased slightly for treated females (− 0.22 ± 0.68 mmol/L [*n* = 13]).

##### Body fat mass

Baseline body fat mass (%) was higher in females (controls: 32.98 ± 6.22 [*n* = 5]; treated: 36.81 ± 6.86 [*n* = 24]) than males (controls: 27.69 ± 8.68 [*n* = 11]; treated: 26.02 ± 6.55 [*n* = 47]). Absence of treatment was associated with an increase in body fat (mean change from baseline: female: + 0.85% ± 1.06% [*n* = 11]; male + 3.46% ± 3.07% [*n* = 47]), whereas GH treatment was associated with a decrease (mean change from baseline: female: − 2.29% ± 4.14% [*n* = 15]; male: − 1.93% ± 4.52% [*n* = 27]).

##### IGF-I SDS

**Fig. 2:**
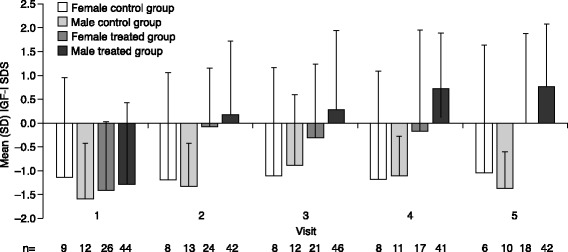
Mean (SD) change in IGF-I SDS from baseline to follow-up visits by gender IGF-I, insulin-like growth factor 1; SD, standard deviation; SDS, standard deviation score.

The increase in GET score was accompanied by an increase in IGF-I SDS in the GH-treated groups. Mean IGF-I SDS was below zero (− 1.13 to − 1.58) for all groups at baseline. At visit 2, change from baseline for treated females was + 1.37 ± 1.14 (*n* = 23), and for treated males was + 1.42 ± 1.21 (*n* = 39) (Fig. 2). The increased level of IGF-I SDS in the treated groups was maintained throughout the study. IGF-I SDS did not substantially change for the control group, remaining below zero at every visit.

## Discussion

This study aimed to design and evaluate an experimental score that integrates the different features of AGHD and demonstrates the pleiotropic therapeutic effects of GH substitution in patients with AGHD. The GET score, which was designed based on evidence in the literature [2, 3], weights items according to their clinical relevance as considered by the study group. Importantly, the GET score can potentially be applied and calculated by physicians in everyday clinical practice.

However, our non-interventional study has shown that in a considerable proportion of patients the GET score at baseline could not be calculated due to missing data. This was surprising since the score was designed so that a GET score could be calculated with a total weighting of variables of only 70%, to account for potential missing data in a real world setting. The mean baseline GET score in GH-untreated patients was ~ 50, with a variation equivalent to ~ 20 points (SD). The contribution for each individual item to the observed GET scores was appropriately spread across the score range (i.e., 0–10 or 0–20, depending on the weighting) and mean scores were positioned, as expected, around the midpoint of the scale, indicating that the predefined score calculation was suitable. The descriptive statistics of the GET score in a GH treatment naïve situation at baseline yielded results within the expected range, indicating that the assumptions made when designing the score were appropriate.

In our proof of concept study, the GET score was evaluated and used to estimate the combined pleiotropic effects of GH by calculating the overall difference in GET score in the full follow-up period between the untreated versus treated group. This required the use of a repeated measures model to account for correlation of data within the individual patient, to handle missing and thus variable numbers of observations, and to detect any overall differences between mean GET scores of the two groups in the full study period. Due to the longitudinal nature of the study, interpretation of the study should be based on the overall difference between the two groups, as estimated by the model and shown in Fig. 1.

Based on the full follow-up period of the study, the GET score was statistically significantly different overall between untreated and GH-treated patients in the expected range of ~ 10 points, representing a clinically relevant difference in HRQoL and/or somatic parameters. This difference was largely driven by the deterioration in GET score over time in the untreated group. The difference reached the magnitude expected, confirming that the assumptions made when designing the score were appropriate. This allows us to state that the GET score may be a suitable instrument to quantify the effects of GH treatment in patients with AGHD in an integrative way.

The GET score includes clinically relevant parameters, weighted according to potential impact on the individual patient. Patients with AGHD have reported the negative impact of their condition on many aspects of daily life [23], and the importance of HRQoL is recognised in clinical practice guidelines [24, 25]. Hence, the HRQoL parameters provide the highest overall contribution to the GET score (40%). Although the sample size of this study is limited, the results are consistent with available literature showing that GH substitution can positively influence HRQoL [26, 27].

BMD contributed significantly to the GET score (weighted 20%); however, unfortunately only a small number of patients in this study underwent DXA analysis, making interpretation of the data difficult. Many patients lacked BMD data because data collection was based on routine clinical practice and not a study protocol. During this study, BMD assessed by DXA z-score did not change significantly. Davidson et al. [28] demonstrated that clinically significant changes in BMD are observed after treatment duration of at least 18–24 months. This limitation could be addressed by studying a larger cohort over a longer period.

IGF-I serum levels are used for GH dose titration [24, 25] and provide an indication of the efficacy of GH therapy and patients’ adherence with treatment. In this study, IGF-I SDS increased from baseline to near zero by visit 2 following GH treatment initiation; this level was maintained or increased throughout the study in the treated groups.

The main limitation of this proof-of-concept study was the observational, non-interventional design, which lacked the methodological rigour of a randomised controlled trial. The control group had a low number of patients and there were also differences between groups; it is likely that, as the decision for treatment was based on physician opinion, the two groups (control and treated) were not homogeneous, with differences in co-morbidities and use of concomitant medication. Treatment adherence was not evaluated in the treatment group, which may have affected the results.

As with many observational studies, missing data were a challenge, and incomplete data sets had to be handled by an appropriate statistical approach. The set of parameters chosen for the GET score was based on published evidence; however, in contrast to the published recommendations [3] these parameters do not seem to be routinely assessed when GH-treatment is warranted in AGHD. The small number of BMD examinations was unexpected for the study group. Potentially, the study duration was too short for BMD follow-up examinations. The fact that a baseline GET score could only be calculated in 75/106 patients is a concern regarding the ability of the GET score to be used in everyday clinical practice. The process of calculating the GET score, particularly the points for SF-36, is laborious, thus limiting the use in routine clinical practice. Nevertheless, an individual comparison of GET scores at baseline and after a period of GH treatment might be of clinical relevance for the assessment of individual clinical response to GHT.

## Conclusions

The newly developed GET score appeared to be a suitable instrument to summarise the features of AGHD and evaluate the pleiotropic response to GH substitution therapy in an integrated way. We suggest the GET score as a tool for clinical studies rather than for routine clinical practice. A further study in a larger cohort and over a longer period of time could overcome some of the shortcomings seen in this project.

## Additional file

Additional file 1: Table S1. GET score point allocation for HRQoL parameters (which comprise 50 of the total of 100 points of the GET score). Table S2 Example of a calculation of SF-36 GET score component points. Table S3 GET score point allocation for somatic parameters (which comprise 50 of the total of 100 points of the GET score).Table S4 Example of calculation of GET score including SF-36 subtotal and addition to other components of the score. GET score calculation. Table S5 Example of calculation of an adjusted GET score due to a missing value (DOCX 39 kb)

## Abbreviations

AGHD: Adults with growth hormone deficiency
BMD: Bone mineral density
DXA: Dual-energy X-ray absorptiometry
EBM: Evidence-based medicine
EQ-5D: EuroQol five dimensions questionnaire
EQ-5D-VAS: VAS component of EQ-5D
GET score: Growth hormone deficiency and Efficacy of Treatment score
GH: Growth hormone
GHD: Growth hormone deficiency
HRQoL: Health-related quality of life
IGF-I: Insulin-like growth factor I
LDL-C: Low-density lipoprotein cholesterol
LSM: Least squares means
SD: Standard deviation
SDS: Standard deviation score
SF-36: Short-Form Health Survey 36
VAS: Visual analogue scale
