# Supplementary material for: Design of the Growth hormone deficiency and Efficacy of Treatment (GET) score and non-interventional proof of concept study
Source: BMC Endocr Disord. 2018 Feb 13;18:10. doi: 10.1186/s12902-018-0237-3 (PMC5810096; doi:10.1186/s12902-018-0237-3)
Supplement: Supplementary file 1 — GET score point allocation for HRQoL parameters (which comprise 50 of the total of 100 points of the GET score). Table S2 Example of a calculation of SF-36 GET score component points. Table S3 GET score point allocation for somatic parameters (which comprise 50 of the total of 100 points of the GET score).Table S4 Example of calculation of GET score including SF-36 subtotal and addition to other components of the score. GET score calculation. Table S5 Example of calculation of an adjusted GET score due to a missing value (DOCX 39 kb) [file 12902_2018_237_MOESM1_ESM.docx]

**Additional File 1**

**Design of the Growth hormone deficiency and Efficacy of Treatment (GET) score and non-interventional proof of concept study**

Peter H. Kann^1^, Simona Bergmann^1^, Martin Bidlingmaier^2^, Christina Dimopoulou^3^, Birgitte T. Pedersen^4^, Günter K. Stalla^3^, Matthias M. Weber^5^, Stefanie Meckes-Ferber^6^

^1^Endocrinology & Diabetology, Philipp’s University Hospital, 35033 Marburg, Germany

^2^Endocrine Laboratory, Medizinische Klinik und Poliklinik IV, Ludwig-Maximilians University, 80336 Munich, Germany

^3^Neuroendocrinology, Max-Planck-Institute for Psychiatry, 80804 Munich, Germany

^4^Epidemiology, Novo Nordisk A/S, 2860 Søborg, Denmark

^5^Endocrinology & Metabolism, Johannes Gutenberg University Hospital, 55131 Mainz, Germany

^6^Clinical, Medical & Regulatory Department, Novo Nordisk Pharma GmbH, 55127 Mainz, Germany

*Correspondence:*

Peter Herbert Kann, MD PhD PhD MA, Professor of Medicine

Division of Endocrinology & Diabetology, Philipp’s University Marburg

Email: [kannp@med.uni-marburg.de](mailto:kannp@med.uni-marburg.de)

**Supporting Information**

**Methods**

***Growth hormone deficiency and Efficacy of Treatment (GET) score assessment and definition***

Allocation of the GET score points from individual HRQoL parameters is given in Supplementary Table 1. Calculation of the SF-36 GET score component points is given in Supplementary Table 2. Allocation of the GET score points from somatic parameters is provided in Supplementary Table 3. Calculation of other components of the GET score and addition to SF-36 subtotal to determine a final GET score when all parameters are available are given in Supplementary Table 4. Calculation of the GET score including a missing value is given in Supplementary Table 5.

**Supplementary Table 1** GET score point allocation for HRQoL parameters (which comprise 50 of the total of 100 points of the GET score)

| **GET score points** | **HRQoL parameter** | | | | | | | | | | | | | | | | | | | |
| --- | --- | --- | --- | --- | --- | --- | --- | --- | --- | --- | --- | --- | --- | --- | --- | --- | --- | --- | --- | --- |
|  | **SF-36*** | | | | | | | | | | | | | | | | **EQ-5D Visual Analogue Scale** | | **Disease-related days off work during the preceding 6 months** | |
|  | **Bodily pain** | | **Emotional role functioning** | | **General health perceptions** | | **Mental health** | | **Physical functioning** | | **Physical role functioning** | | **Social functioning** | | **Vitality** | |  |  |  |  |
|  | Min | Max | Min | Max | Min | Max | Min | Max | Min | Max | Min | Max | Min | Max | Min | Max | Min | Max | Min | Max |
| 0 | <48 | 48 | <15 | 15 | <38 | 38 | <48 | 48 | <42 | 42 | <31 | 31 | <48 | 48 | <23 | 23 | <40 | 40 | 31 | >31 |
| 1 | 49 | 50 | 16 | 20 | 39 | 40 | 49 | 50 | 43 | 45 | 32 | 35 | 49 | 51 | 24 | 26 | 41 | 43 | 28 | 30 |
| 2 | 51 | 52 | 21 | 25 | 41 | 42 | 51 | 52 | 46 | 48 | 36 | 39 | 52 | 54 | 27 | 29 | 44 | 46 | 25 | 27 |
| 3 | 53 | 54 | 26 | 30 | 43 | 44 | 53 | 54 | 49 | 51 | 40 | 43 | 55 | 57 | 30 | 32 | 47 | 49 | 22 | 24 |
| 4 | 55 | 56 | 31 | 35 | 45 | 46 | 55 | 56 | 52 | 54 | 44 | 47 | 58 | 60 | 33 | 35 | 50 | 52 | 19 | 21 |
| 5 | 57 | 58 | 36 | 40 | 47 | 48 | 57 | 58 | 55 | 57 | 48 | 51 | 61 | 63 | 36 | 38 | 53 | 55 | 16 | 18 |
| 6 | 59 | 60 | 41 | 45 | 49 | 50 | 59 | 60 | 58 | 60 | 52 | 55 | 64 | 66 | 39 | 41 | 56 | 58 | 13 | 15 |
| 7 | 61 | 62 | 46 | 50 | 51 | 52 | 61 | 62 | 61 | 63 | 56 | 59 | 67 | 69 | 42 | 44 | 59 | 61 | 10 | 12 |
| 8 | 63 | 64 | 51 | 55 | 53 | 54 | 63 | 64 | 64 | 66 | 60 | 63 | 70 | 72 | 45 | 47 | 62 | 64 | 7 | 9 |
| 9 | 65 | 66 | 56 | 60 | 55 | 56 | 65 | 66 | 67 | 69 | 64 | 67 | 73 | 75 | 48 | 50 | 65 | 67 | 4 | 6 |
| 10 | 67 | 68 | 61 | 65 | 57 | 58 | 67 | 68 | 70 | 72 | 68 | 71 | 76 | 78 | 51 | 53 | 68 | 70 | <3 | 3 |
| 11 | 69 | 70 | 66 | 70 | 59 | 60 | 69 | 70 | 73 | 75 | 72 | 75 | 79 | 81 | 54 | 56 | 71 | 73 | ─ | ─ |
| 12 | 71 | 72 | 71 | 75 | 61 | 62 | 71 | 72 | 76 | 78 | 76 | 79 | 82 | 84 | 57 | 59 | 74 | 76 | ─ | ─ |
| 13 | 73 | 74 | 76 | 80 | 63 | 64 | 73 | 74 | 79 | 81 | 80 | 83 | 85 | 87 | 60 | 62 | 77 | 79 | ─ | ─ |
| 14 | 75 | 76 | 81 | 85 | 65 | 66 | 75 | 76 | 82 | 84 | 84 | 87 | 88 | 90 | 63 | 65 | 80 | 82 | ─ | ─ |
| 15 | 77 | 78 | 86 | 90 | 67 | 68 | 77 | 78 | 85 | 87 | 88 | 91 | 91 | 93 | 66 | 68 | 83 | 85 | ─ | ─ |
| 16 | 79 | 80 | 91 | 95 | 69 | 70 | 79 | 80 | 88 | 90 | 92 | 95 | 94 | 96 | 69 | 71 | 86 | 88 | ─ | ─ |
| 17 | 81 | 82 | 96 | 100 | 71 | 72 | 81 | 82 | 91 | 93 | 96 | 99 | 97 | 99 | 72 | 74 | 89 | 91 | ─ | ─ |
| 18 | 83 | 84 | 101 | 105 | 73 | 74 | 83 | 84 | 94 | 96 | 100 | 103 | 100 | 102 | 75 | 77 | 92 | 94 | ─ | ─ |
| 19 | 85 | 86 | 106 | 110 | 75 | 76 | 85 | 86 | 97 | 99 | 104 | 107 | 103 | 105 | 78 | 80 | 95 | 97 | ─ | ─ |
| 20 | 87 | >87 | 111 | >111 | 77 | >77 | 87 | >87 | 100 | >100 | 108 | >108 | 106 | >106 | 81 | >81 | 98 | >98 | ─ | ─ |

*SF-36 accounts for 20% of the total GET score. The sum of all the SF-36 scores is divided by the number of dimensions to give a SF-36 GET score component in the range 0–20. See example in Supplementary Table 2.

EQ-5D, EuroQol five dimensions questionnaire; GET, Growth hormone deficiency and Efficacy of Treatment; SF-36, Short-Form Health Survey 36.

**Supplementary Table 2** Example of a calculation of SF-36 GET score component points

| **Dimension** | **Transformed SF-36 domain score^a^** | **GET score point** |
| --- | --- | --- |
| Bodily pain | 100 | 20 |
| Emotional role functioning | 50 | 7 |
| General health perceptions | 47 | 5 |
| Mental health | 65 | 9 |
| Physical functioning | 90 | 16 |
| Physical role functioning | 62.5 | 8 |
| Social functioning | 75 | 9 |
| Vitality | 37.5 | 5 |
| Sum | ─ | 79 |
| SF-36 GET score component (sum divided by 8) | ─ | 9.875 |

**^a^**For transformation of the SF-36 score refer to SF-36 manual [1].

GET, Growth hormone deficiency and Efficacy of Treatment; SF-36, Short-Form Health Survey 36.

**Supplementary Table 3** GET score point allocation for somatic parameters (which comprise 50 of the total of 100 points of the GET score)

| **Somatic parameters** | | | | | | | | | | |
| --- | --- | --- | --- | --- | --- | --- | --- | --- | --- | --- |
| **GET score points** | **Bone mineral density (z-score)** | | **Female waist circumference (cm)** | | **Male waist circumference (cm)** | | **LDL cholesterol (mg/dL)** | | **Body fat mass (%)** | |
|  | Min | Max | Min | Max | Min | Max | Min | Max | Min | Max |
| 0 | <–2.00 | –2.00 | 99 | >99 | 113 | >113 | 155 | >155 | 44.1 | >44.1 |
| 1 | –1.99 | –1.90 | 97 | 98 | 111 | 112 | 149 | 154 | 41.6 | 44.0 |
| 2 | –1.89 | –1.80 | 95 | 96 | 109 | 110 | 143 | 148 | 39.1 | 41.5 |
| 3 | –1.79 | –1.70 | 93 | 94 | 107 | 108 | 137 | 142 | 36.6 | 39.0 |
| 4 | –1.69 | –1.60 | 91 | 92 | 105 | 106 | 131 | 136 | 34.1 | 36.5 |
| 5 | –1.59 | –1.50 | 89 | 90 | 103 | 104 | 125 | 130 | 31.6 | 34.0 |
| 6 | –1.49 | –1.40 | 87 | 88 | 101 | 102 | 119 | 124 | 29.1 | 31.5 |
| 7 | –1.39 | –1.30 | 85 | 86 | 99 | 100 | 113 | 118 | 26.6 | 29.0 |
| 8 | –1.29 | –1.20 | 83 | 84 | 97 | 98 | 107 | 112 | 24.1 | 26.5 |
| 9 | –1.19 | –1.10 | 81 | 82 | 95 | 96 | 101 | 106 | 21.6 | 24.0 |
| 10 | –1.09 | –1.00 | <80 | 80 | <94 | 94 | <100 | 100 | <21.5 | 21.5 |
| 11 | –0.99 | –0.90 | ─ | ─ | ─ | ─ | ─ | ─ | ─ | ─ |
| 12 | –0.89 | –0.80 | ─ | ─ | ─ | ─ | ─ | ─ | ─ | ─ |
| 13 | –0.79 | –0.70 | ─ | ─ | ─ | ─ | ─ | ─ | ─ | ─ |
| 14 | -0.69 | –0.60 | ─ | ─ | ─ | ─ | ─ | ─ | ─ | ─ |
| 15 | –0.59 | –0.50 | ─ | ─ | ─ | ─ | ─ | ─ | ─ | ─ |
| 16 | –0.49 | –0.40 | ─ | ─ | ─ | ─ | ─ | ─ | ─ | ─ |
| 17 | -0.39 | –0.30 | ─ | ─ | ─ | ─ | ─ | ─ | ─ | ─ |
| 18 | –0.29 | –0.20 | ─ | ─ | ─ | ─ | ─ | ─ | ─ | ─ |
| 19 | -0.19 | –0.10 | ─ | ─ | ─ | ─ | ─ | ─ | ─ | ─ |
| 20 | –0.09 | >–0.09 | ─ | ─ | ─ | ─ | ─ | ─ | ─ | ─ |

GET, Growth hormone deficiency and Efficacy of Treatment; LDL, low-density lipoprotein.

***GET score calculation***

To calculate a GET score, a set of variables (eight SF-36 domain items, disease-related days off work, bone mineral density [BMD], waist circumference, LDL cholesterol and body fat mass and EQ-5D Visual Analogue Scale) is required.

The first step is to calculate the SF-36 GET score points based on the transformed SF-36 domain score for all eight SF-36 domains (see Supplementary Table 2) and the overall SF-36 GET score points is subsequently calculated by taking the average of all eight SF-36 GET score points. The next step is to look up the GET score points for the remaining variables using the GET score points as shown in Supplementary Tables 1 and 3. Addition of the SF-36 GET score points to the sum of the other GET score points comprises the final GET score.

A worked example of a calculation of the GET score based on a full set of variables is given in Supplementary Table 4. The final GET score for this full set of variables is 67.875.

**Supplementary Table 4** Example of calculation of GET score including SF-36 subtotal and addition to other components of the score

| **Relevant parameter** | **Measurement performed** | **GET score points** |
| --- | --- | --- |
| SF-36 GET score component* | 9.875 | 9.875 |
| EQ-5D visual analogue scale | 60 | 7 |
| Disease-related days off work | 0 | 10 |
| Bone mineral density | 1.1 | 20 |
| Waist circumference | 100 | 7 |
| LDL cholesterol | 107 | 8 |
| Body fat mass | 29.4 | 6 |
| Determined GET Score | ─ | 67.875 |

*See Supplementary Table 2

EQ-5D, EuroQol five dimensions questionnaire; GET, Growth hormone deficiency and Efficacy of Treatment; LDL, low-density lipoprotein; SF-36, Short-Form Health Survey 36

If a full set of variables are not available an adjusted GET score can be calculated. A minimum number of parameters giving a total weighting of ≥70% is required to calculate an adjusted GET score. If the available items comprise a weighting <70, the GET score cannot be calculated. If the weightings are between 70 and 90, the score must be recalculated as follows:

$$\frac{determined score*100}{maximum achievable score for the available set of variables}$$

A worked example of a calculation of the GET score, when a parameter is missing is given in Supplementary Table 5.

**Supplementary Table 5** Example of calculation of an adjusted GET score due to a missing value

| Parameter | Weight | GET score points |
| --- | --- | --- |
| SF-36 GET score component | 20 | 20 |
| EQ-5D visual analogue scale | 20 | 20 |
| Disease-related days off work | 10 | 7 |
| Bone mineral density | 20 | Missing |
| Waist circumference | 10 | 5 |
| LDL cholesterol | 10 | 10 |
| Body fat mass | 10 | 5 |
|  | ─ |  |
| Determined GET score |  | 67 |
| Adjusted GET score | $\frac{67*100}{80}$ | 83.75 |

EQ-5D, EuroQol five dimensions questionnaire; LDL, low-density lipoprotein; SF-36, Short-Form Health Survey 36.

**References**

1 Ware JE Jr. SF-36 Health Survey Update. http://www.sf-36.org/tools/SF36.shtml (2007). Accessed July 2017.
